# Application of Nanoparticle Technologies in the Combat against Anti-Microbial Resistance

**DOI:** 10.3390/pharmaceutics10010011

**Published:** 2018-01-14

**Authors:** Mayur Kumar, Anthony Curtis, Clare Hoskins

**Affiliations:** School of Pharmacy, Institute of Science and Technology for Medicine, Keele University, Keele, Staffordshire ST5 6DB, UK; w2v11@students.keele.ac.uk (M.K.); a.d.m.curtis@keele.ac.uk (A.C.)

**Keywords:** anti-microbial resistance, anti-bacterial modality, drug carriers, nanoparticles, oxidative stress

## Abstract

Anti-microbial resistance is a growing problem that has impacted the world and brought about the beginning of the end for the old generation of antibiotics. Increasingly, more antibiotics are being prescribed unnecessarily and this reckless practice has resulted in increased resistance towards these drugs, rendering them useless against infection. Nanotechnology presents a potential answer to anti-microbial resistance, which could stimulate innovation and create a new generation of antibiotic treatments for future medicines. Preserving existing antibiotic activity through novel formulation into or onto nanotechnologies can increase clinical longevity of action against infection. Additionally, the unique physiochemical properties of nanoparticles can provide new anti-bacterial modes of action which can also be explored. Simply concentrating on antibiotic prescribing habits will not resolve the issue but rather mitigate it. Thus, new scientific approaches through the development of novel antibiotics and formulations is required in order to employ a new generation of therapies to combat anti-microbial resistance.

## 1. Introduction

**Figure 1:**
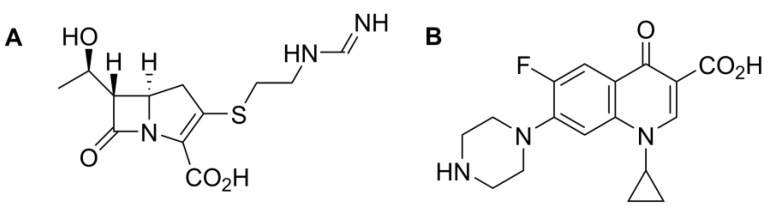
Chemical structure of (**A**) imipenem and (**B**) ciprofloxacin.

Anti-microbial resistance is a global problem that is affecting modern healthcare. Due to inappropriate habits in the prescribing of antibiotics, anti-microbial resistance to an array of different classes of antibiotics has occurred [1]. This phenomenon will undoubtedly significantly impact the future efficacy and usage of antibiotics within both community and hospital care globally [2,3]. In February 2017, the World Health Organisation (WHO) published its first-ever list of antibiotic-resistant pathogens for which new anti-microbials are needed urgently [4]. Out of the 12 resistant pathogens listed, seven were noted to possess resistance to beta-lactam antibiotics. The three pathogens categorised as “Critical” are resistant to carbapenems, imipenem for example, and four others are resistant to fluoroquinolones, such as ciprofloxacin (Figure 1) which are also widely used in clinical practice. This is an alarming fact as in the future this will not only challenge prescribing practices but will also increase the difficulty in obtaining suitable antibiotics to treat patients. Nevertheless, the WHO has commented that this is an opportunity for the research and development (R & D) sector to produce novel antibiotics, setting a new goal for future research strategies. 

**Figure 2:**
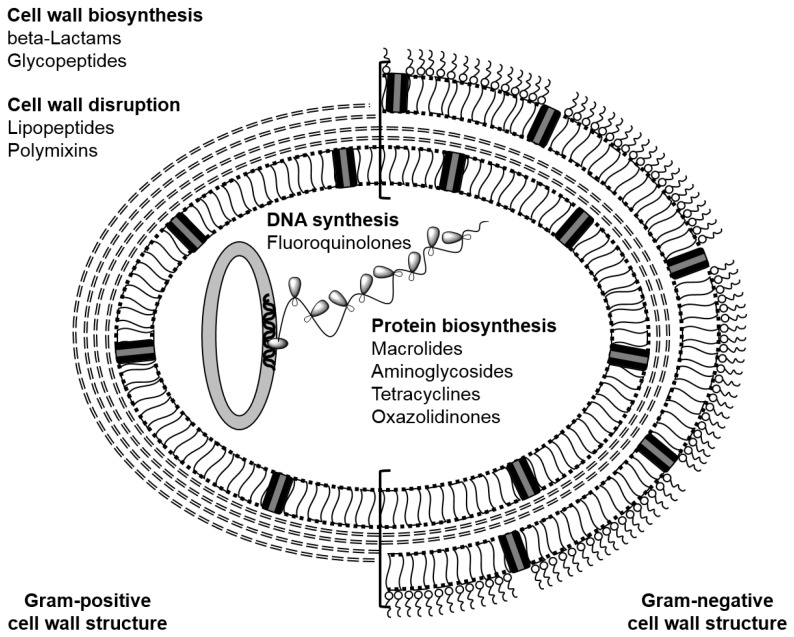
Common antibiotic classes and their targets.

Bacteria are prokaryotes, they do not possess a nuclear membrane and are classified as being Gram-positive or Gram-negative, based broadly upon the structure of their cell wall (Figure 2). Gram’s stain experiment classifies bacteria according to the ability of the bacterial cell wall to absorb and retain crystal violet dye: Gram-positive bacteria retain the stain whereas Gram-negative bacteria do not [5]. Gram-positive bacteria possess a rigid cell wall comprising a thick layer of peptidoglycan, which is composed of carbohydrate polymers cross-linked through peptide residues [6]. Teichoic acid is found on the surface of Gram-positive bacteria, which confers the ability to chelate metal ions and act as a protection mechanism against the host immune response [7]. Lipoteichoic acids are also present in the cell membrane, which allow surface adherence [8]. Conversely, Gram-negative bacteria contain a thinner, more rigid peptidoglycan layer with much shorter cross-links, surrounded by a lipid membrane with lipopolysaccharides (LPS) presented on the surface [9]. *Staphylococcus aureus* is an example of a Gram-positive bacterium: Methicillin resistant *S. aureus* (MRSA) [10] is commonly recognised by the lay person as being associated with antibiotic resistance. MRSA infections require prolonged treatment regimens, often with powerful antibiotics and are consequently responsible for elevated levels of patient hospitalisation and public spending. *Klebsiella pneumoniae* is a Gram-negative bacterium that is present normally in the gastrointestinal tract, along with other species of bacteria, but is associated with pneumonia, urinary tract and wound infections, bacteremia and septicaemia [11]. Like many examples of Enterobacteriaceae, *K. pneumoniae* has developed resistance to carbapenems [12]. Mycobacteria present an additional threat: Mycobacteria have a unique, hydrophobic cell wall structure and latent asymptomatic infections are common. A restricted number of antibiotics are reserved for treatment of *Mycobacterium tuberculosis* infection (TB) but resistance to these is on the increase. TB was declared a “global emergency” by the WHO [13], extensively drug-resistant TB (XDR-TB) is now rife and totally drug-resistant strains of *M. tuberculosis* have been observed [14].

The majority of common antibiotics discovered in the 20th century during the “golden age” are derived from natural products. Antibiotics can be classified by their target, their molecular structure and mode of action. For example, the principal targets for antibiotics in bacteria are assembly of the cell wall, protein synthesis and the synthesis of nucleic acids. Classes of antibiotics which inhibit the synthesis of peptidoglycan include the beta-lactams and glycopeptides; these two classes have different modes of action so their use does not confer cross-resistance, though the glycopeptides are only of use to treat Gram-positive infections. Major classes of antibiotics that target protein synthesis include the macrolides and aminoglycosides. Synthesis and replication of DNA is inhibited by the fluoroquinolones [15]. Other antibiotics, such as the lipopeptides and polymixins can disrupt the cell walls of bacteria: colistin, a polymixin, is often cited as the drug of last resort yet resistance to colistin has been observed [16]. Significantly, none of the classes of antibiotics used clinically incorporate metal atoms in their structure.

The over-use of potent antibiotics such as vancomycin, a glycopeptide that inhibits the formation of cross-links in the assembly of peptidoglycan, to treat infections by pathogenic Gram-positive bacteria which are resistant to beta-lactams has led to a greater occurrence of resistance to glycopeptides in these species [17]. This further depletes the pool of last line antibiotics that are useful clinically. Intrinsic resistance mechanisms exhibited by bacteria and coded for in the genome, examples of which include a lack of oxidative metabolism which prevents drug uptake or the presence of an outer lipid membrane in the Gram-negative bacteria cell wall which prevents uptake of glycopeptides, present other challenges. Resistance to antibiotics can be acquired [18,19] by horizontal gene transfer whereby antibiotic-resistant bacteria donate DNA, typically integrated into a plasmid and codes for that resistance mechanism, to previously susceptible bacteria. This DNA is retained in the recipient cell, within a plasmid or is transposed into the genome, and expression results in organisms that now harbour resistance to the given antibiotic. This may include the acquisition of code for the production of beta-lactamases that are responsible for enzymic degradation of beta-lactam antibiotics [20], or the transfer of transposable elements such as the transposon Tn1546 which confers VanA type resistance to vancomycin in the Enterococci and in *S. aureus* [21], for example. 

Today, with nanotechnology being more prevalent and applied in medicine it is not surprising to see nanoparticle technologies being utilised in the fight against antibiotic resistance. Nanoparticles can be utilised by (1) amalgamation with existing clinically relevant antibiotics to adapt and enhance their physiochemical properties to overcome anti-microbial resistance mechanisms, or (2) as anti-microbial agents in themselves, the colloidal forms of silver, zinc, copper, titanium and vanadium for example. As noted above, the three principal targets for antibiotic action are inhibition of synthesis or disruption of the cell wall, translation and transcription during protein synthesis and the synthesis of nucleic acids. However, nanoparticle technologies have been found to affect the bacterial respiration system, inducing generation of reactive oxygen species (ROS) in bacteria via compromising the bacterial antioxidant system. This presents a new therapeutic approach to overcome anti-microbial resistance. Silver nanoparticles, for example, target the bacterial cell wall [22], so by coupling clinically relevant antibiotics onto the surface of these colloids the anti-bacterial activity of the drugs will be enhanced through synergy. In this review, we will report the current development of nanoparticle technology for combatting anti-microbial resistance. 

## 2. Challenges to the Development of New Anti-Microbials

Despite the persistent growth in the occurrence of resistance to anti-microbial agents within bacteria, there is a paucity of new antibiotics entering the market. This is possibly due to the reduced return on investment from such R & D activity, with the prospect of each new antibiotic having a limited market after resistance to that drug has arisen. It has been proposed that an investment of $1.2 billion USD into R & D is required to conduct the extensive research required to deliver a small portfolio of new commercial entities [23]. Alternatively, it is more beneficial financially to develop and market analogues of existing therapies rather than invest in new research [24]. Additionally, international regulatory barriers have affected approval requirements, whereby a compound must exhibit superiority instead of non-inferiority [25] to conventional therapies. Post-marketing pharmacovigilance of adverse events may also jeopardise any potential candidates or therapies and, hence, the sector has not prioritised research into novel antibiotics. This places the future of antibiotic drug discovery in jeopardy: We are experiencing the situation in which some agencies demand development of a new antibiotic arsenal, while at the same time other agencies inhibit such development, resulting in stalemate. The pharmaceutical industry plays a vital role in driving R & D into new antibiotics. Currently, there are few companies that invest significant resources in research and development of antibiotics, with many others concentrating on developing generics as there is low risk in not gaining return on investment compared to developing novel compounds [26]. Equally, most big pharmaceutical companies have prioritised investment into drugs associated with chronic diseases that require prolonged therapies [1]. In bacterial infection, therapy is mostly acute and, hence, development of novel therapies has not drawn much attention when compared with other markets, such as oncology where patient prognosis over a longer timeframe has demanded new therapies and overshadowed the growing anti-microbial resistance epidemic. Interestingly, 50% of all antibiotics developed in “the golden age” are still used clinically, with few new classes of antibiotics being developed in the 21st Century [27]. Those novel antibiotics coming through the pipeline are typically analogues of previous generations of antibiotics, which retain the same mechanism of action. Examples of antibiotics currently emerging from the pipeline are gepotidacin (GSK2140944), a type 2 topoisomerase inhibitor with a mode of action which differs from the commonly prescribed fluoroquinolones [28], and GSK3342830 which is a cephalosporin with activity against Gram-negative infections [29].

## 3. Challenges to the Strategies Employed to Develop New Anti-Microbials

**Figure 3:**
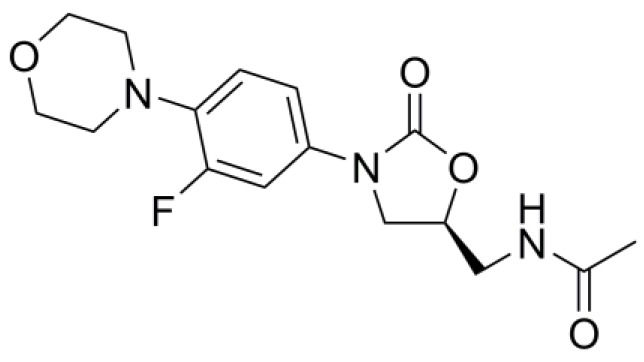
Chemical structure of linezolid.

Since the discovery of the naturally occurring penicillin in 1928 by Alexander Fleming, modification of the core penicillin molecular structure by altering the properties of the side chain to give semi-synthetic penicillins has been the major strategy employed to develop new beta-lactam antibiotics from the penicillin family [30]. Besides the penicillins, development of other classes of antibiotics from naturally occurring compounds has adopted the same strategy. Research using modern medicinal chemistry paradigms has had limited success in developing novel, synthetic anti-microbial agents that have novel modes of action to exert their anti-microbial properties. For example, development of the oxazolidinone class of antibiotics, linezolid for example (Figure 3), in the latter decades of the 20th Century represents a recent example of a novel antibiotic class with a unique mechanism of action. However, in common with the other classes of antibiotics available clinically, a similar fate has befallen the oxazolidinones in that there is an increase in treatment failure due to the incidence of bacterial resistance to the oxazolidinones. The controlled use of more established classifications of antibiotics is often used as a strategy to combat resistance in bacteria [31]. However, a problem that is faced when using some established classes of antibiotics is their toxicity: As examples, a significant number of patients are allergic to penicillin, fluoroquinolones must not be used in children or elderly patients due to the risk of musculoskeletal damage and tendinopathy, and dose-related progressive ototoxicity is a risk when prescribing aminoglycosides. Nevertheless, there are potential opportunities to develop novel nanoparticle formulations to increase the efficacy of established classes of antibiotics, thus enabling dose reduction and limiting the associated toxicity. 

## 4. Nanotechnology in Biomedicine 

Nanotechnology is used widely in today’s biomedicine, with applications in drug delivery. It is estimated that 60% of all drug entities under development are practically insoluble: solubility is a major challenge in achieving desired bioavailability and appropriate levels of drug efficacy [32]. Nanotechnologies have been developed which not only improve drug solubility via encapsulation but also promote enhanced permeation of membranes, longer circulation times and greater overall efficiency. As knowledge has grown in this field and a multidisciplinary approach employed, targeted therapeutics have been developed which allow the drug of choice to reach the desired site of action in the body, thus allowing many toxic, insoluble and non-permeable drugs to enter clinical trial as potential therapies [33].

## 5. Nanoparticles as Anti-Microbial Agents

### 5.1. Synergistic Application of Nanoparticles with Antibiotics

**Figure 4:**
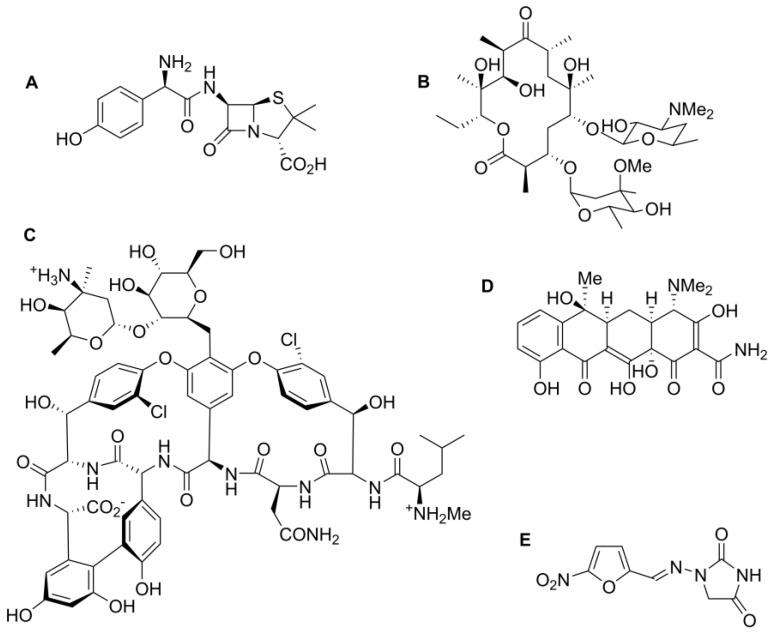
Chemical structures of (**A**) amoxicillin; (**B**) erythromycin; (**C**) vancomycin; (**D**) tetracycline and (**E**) nitrofurantoin.

The conventional chemotherapeutic method of fighting infections with small-molecule antibiotic drugs has led to the current, significant challenge of resistant bacteria. A particular problem is the evolution of bacteria that are resistant to many antibiotic classes, so-called multidrug-resistant (MDR) bacteria. As the number of useful first line antibiotics diminishes and last line drugs are increasingly used to combat resistant infections, the number of treatment options for patients reduces. Reserving the use of last line antibiotics will only delay the inevitable evolution of resistance to these antibiotics. Conjugation of small molecule antibiotic drugs onto nanoparticles, silver nanoparticles for example, is a possible approach to overcome the challenge of bacterial resistance by exploiting the synergistic effect observed in the use of both the drug and the nanoparticle together. Shahverdi and colleagues demonstrated elevated antibiotic activity against a panel of bacteria which included *S. aureus* and *Escherichia coli*, using a combination treatment of silver nanoparticles with established antibiotics [34]. Studies using colloidal silver conjugated with widely used antibiotics, such as amoxicillin, erythromycin and vancomycin, itself only active against Gram-positive bacteria, (Figure 4) have shown enhanced anti-bacterial activity [34]. Furthermore, Deng and colleagues showed that a combination of tetracycline (Figure 4) bound to the surface of silver nanoparticles resulted in elevated anti-bacterial action against *Salmonella typhimurium* relative to silver nanoparticles alone [35]. The tetracycline-silver nanoparticle complex enabled increased drug action due to greater silver accumulation around the cell and, hence, this enhanced contact with the bacterial cell wall resulted in increased bacterial growth inhibition. Similarly, Banoee and colleagues demonstrated an increase in synergistic anti-bacterial activity using zinc oxide and ciprofloxacin (Figure 1) against *E. coli* and *S. aureus* [36]. Despite achieving synergistic activity with ciprofloxacin, it was noted that combination treatment of zinc oxide nanoparticles with amoxicillin and with nitrofurantoin (Figure 4) in fact decreased the anti-bacterial activity against *E. coli* and *S. aureus*. Consequently, this study demonstrates that combination treatment using nanoparticles combined with any antibiotic does not always result in increased anti-microbial activity and that this may be drug- and nanoparticle-dependant [36]. Despite that observation, these findings show potential for increasing the effectiveness and longevity of these traditional antibiotics.

### 5.2. Intrinsic Properties of Nanoparticles Which Confer Activity Against Bacteria

#### 5.2.1. Generation of Reactive Oxidative Species

**Figure 5:**
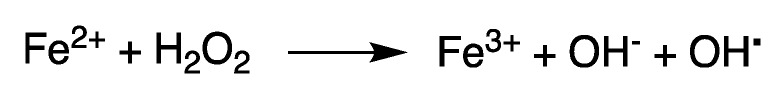
Fenton reaction with Fe in its divalent form.

Reactive oxygen species (ROS) are formed in bacteria from aerobic respiration. Additionally, free radicals can be generated via exposure to UV irradiation [37]. Derivatives generated by aerobic respiratory metabolism, such as hydrogen peroxide, superoxide, and hydroxyl radicals are very harmful and can lead to oxidative stress, which in turn causes damage to nucleotides and lipids in the prokaryote cell [38]. By considering the function of ROS in bacterial respiration, these derivatives can be used to cause bacterial mortality by compromising the cell’s antioxidant defence system. Superoxide dismutase is responsible for bacterial defence against ROS by forming oxygen and hydrogen peroxide by fusion of two superoxide anions [39]. Hence, oxidative stress can be induced by generating ROS via the Fenton reaction, represented in Figure 5. This is achieved by introduction of a divalent metal ion that will react with hydrogen peroxide to produce hydroxyl radicals which are capable of damaging bacterial DNA and lipids [40].

Li and colleagues reported anti-bacterial properties of zinc oxide and titanium oxide nanoparticles after UV irradiation. Both nanoparticles generated superoxide, hydroxyl and singlet oxygen radicals that induce oxidative stress and which resulted in anti-microbial activity [41]. Similarly, Reddy and colleagues found that zinc nanoparticles have anti-bacterial activity via ROS action, initiated by reducing the catalase enzyme synthesis which protects the bacteria form oxidative stress [42]. Furthermore, Dwivedi and colleagues reported the use of zinc nanoparticles which can interact with *Pseudomonas aeruginosa* to generate ROS [43]. They showed that zinc nanoparticles first interact with the outer membrane of the *P. aeruginosa* cell, after which the nanoparticles enter the cell and subsequently cause bacterial damage. The authors hypothesized that intracellular damage was due to ROS production after inhibition of the respiratory enzymes which led to the increase in ROS formation [43]. This hypothesis could be confirmed by inhibition of superoxide dismutase as previously reported by Birben [39] and inhibition of catalase enzyme in the case of Reddy and colleagues [42]. 

Chatterjee and colleagues reported the anti-microbial activity of copper nanoparticles on *E. coli* caused by lipid peroxidation [44]: Oxidative degradation of polyunsaturated lipids in the bacterial membrane by peroxidation resulted in bacterial cell death by augmenting bacterial homeostasis. Disruption to the membrane was also observed when the bacteria were incubated with copper nanoparticles. Similarly, Padmavathy reported the use of zinc oxide nanoparticles capable of ROS generation, resulting in damage to bacterial DNA and cellular proteins [45]. Additionally, these nanoparticles were shown to infringe the bacterial lipid layer of the cell wall that resulted in damage to its structure. By compromising the bacterial lipid layer this leads to exposure of intracellular components of the bacteria that are vital for its homeostasis and proliferation. Thus, by damaging the cell wall this leads to disrupted intracellular homeostasis and compromised bacterial function which causes mortality.

Table 1 shows examples of nanoparticles which possess intrinsic anti-microbial properties across a range of species of bacteria.

#### 5.2.2. Effects of Nanoparticles on Bacterial DNA and Metabolism

Gold nanoparticles were found to exert anti-bacterial activity by affecting prokaryote protein synthesis. Ribosomal tRNA is involved in protein synthesis where it delivers amino acids for translation, in order to synthesize new proteins [52]. Shamaila and colleagues showed that colloidal gold affected protein synthesis by reducing the affinity of the ribosome for tRNA [53]. Furthermore, they showed that the gold hindered bacterial metabolism by decreasing the activity of adenosine triphosphate (ATP) synthase, the enzyme responsible for the formation of ATP from the reaction of inorganic phosphate and ADP [53]. Moreover, the gold was also observed to affect the bacterial respiratory chain by attacking nicotinamide adenine dinucleotide (NADH) dehydrogenase [53]. Here, the gold nanoparticles were found to be irreversibly bound onto the thiol groups present on NADH dehydrogenase, thus affecting the reduction-oxidation balance within the cell which results in the generation of oxidative stress.

### 5.3. Effect of Nanoparticles on Inhibition of Biofilm Formation and Associated Infections

The presence of biofilm is often associated with chronic infections [54] that may have transferred from surfaces, such as contaminated medical equipment and consequently presents problems predominantly in hospitals and other healthcare settings [55]. Biofilm is a robust coating consisting of a collection of bacterial communities which have adhered onto dense surfaces [56]. The biofilm provides an additional resilient barrier against antibiotic treatment for the bacteria which explains why infections caused by biofilm are more difficult to treat and may take longer to present [57,58]. This resilience on abiotic surfaces means the microorganisms have a greater opportunity to spread within a nosocomial setting which is an issue in hospitals especially, as the bacterial infection can be transmitted more easily between patients from medical equipment. Antibiotics used prophylactically during surgery normally decrease the probability of infection. However, since small doses are given to patients for prophylaxis this increases the chance of developing a resistant infection, especially dangerous if this resistance is present in colonies that have adhered to implants. If a biofilm has entered the host this enables the bacteria to evade the host’s immune system which then leads to chronic infection.

Chitosan is a biopolymer which was found to possess anti-bacterial activity when iron oxide nanoparticles were incorporated into its structure. Iron oxide nanoparticles themselves are known to possess intrinsic anti-microbial activity. The mechanism of this activity is the production of ROS by the Fenton reaction, from free radicals produced during oxidation of the iron [59]. Chávez de Paz and colleagues also demonstrated inhibition of biofilm formation and anti-microbial action arising from a chitosan composite [60]. Shi and colleagues reported biofilm inhibition in the presence of chitosan-coated iron oxide nanoparticles in an *S. aureus* model [61]. The proposed explanation for chitosan-coated iron oxide nanoparticles modality was explained by Shrifian-Esfahni [62], whereby positively charged amino groups of chitosan associate with negatively charged modules in the bacterial cell wall, such as *N*-acetyl-muramic acid and sialic acid [62]. By this electrostatic interaction, chitosan possibly limits bacterial growth via enzyme inhibition and metal chelation.

Titanium dioxide nanoparticles have also been found to inhibit biofilm formation. Jesline and colleagues demonstrated inhibition of a MRSA biofilm in the presence of titanium oxide [63]. Application of titanium dioxide as an anti-bacterial agent has good potential since it is approved for use already in medicines and cosmetics, for example. Furthermore, in addition to exhibiting anti-bacterial efficacy in resistant bacteria it is also possible to conjugate antibiotics onto the surface of titanium dioxide nanoparticles to assess whether there us any dual or synergistic anti-bacterial action. Roy and colleagues demonstrated anti-bacterial activity of titanium dioxide nanoparticles conjugated with different classes of antibiotics [64]. They showed elevated anti-bacterial activity as observed by an increase in the zone of inhibition against MRSA which was treated with nanoparticles conjugated to an individual antibiotic from the range investigated. The authors concluded that the effectiveness of the antibiotics used was increased when treating MRSA in the presence of titanium dioxide nanoparticles. However, the action responsible for such improved anti-bacterial activity still needs to be studied [64]. Titanium nanoparticles were also found to be beneficial against MRSA in bone implants. Calcium-Titanium Ca-Ti nanoparticles have been reported to elevate anti-bacterial activity against MRSA infections in rabbits. Cao and colleagues proposed that calcium nanoparticles were responsible for generating ROS from the reaction of calcium with water, producing hydroxyl species which were then responsible for bacterial damage [65]. 

### 5.4. Physiochemical Properties of Nanoparticles Which Influence Anti-Microbial Activity 

#### 5.4.1. Size

Nanoparticle size is key in the interaction with bacterial surfaces as it will dictate the quantity of nanoparticles capable of covering the cell surface, or the degree to which nanoparticles will penetrate the bacterial cell wall. These parameters have a direct correlation to the extent of anti-bacterial activity achieved. The mechanism of action of the nanoparticles, in terms of adhering to the cell wall structures or intracellular internalisation is also dependant on the nanoparticle type. Nanoparticles up to 50 nm diameter possess the ability to penetrate beyond the bacterial cell wall and can target the DNA inside. Morones and colleagues reported the use of silver nanoparticles with a diameter of 10 nm [66]: The particles possessed the ability to penetrate the bacteria and thus result in anti-microbial action [66]. Moreover, anti-microbial activity of silver nanoparticles against *Bacillus subtilis* was reported by Hsueh and colleagues [51], whereby silver nanoparticles with a 10 nm diameter halted *B. subtilis* growth. Besides the size of the nanoparticle, it was not known whether the nanoparticle morphology was also important for efficacy. Sadeghi and colleagues reported the use of silver nanoparticles of different morphologies against *E. coli* and *S. aureus* [67]. They found that regardless of the shape of silver nanoparticles, anti-microbial activity was achieved. Thus, this indicates that nanoparticle morphology may not play a significant role in anti-microbial activity [67]. 

#### 5.4.2. Zeta Potential 

Zeta potential has been observed to play an important role in anti-bacterial activity of nanoparticles. Zeta potential is an important property which contributes to an understanding of the interaction of nanoparticles with bacterial cell surfaces [68]. The zeta potential will determine the potential for nanoparticles to be exploited as drug carriers and also how augmenting bacterial permeability can affect anti-microbial action. Moreover, the zeta potential can dictate the pharmacokinetic behaviour of nanoparticles, especially important due to various pH changes within the body that can affect the charge of the nanoparticle. The zeta potential is especially important in dictating the stability of colloidal nanoparticle formulations. With respect to bacteria, zeta potential can determine how much repulsion or association there will be between the nanoparticle and the bacterial cell surface [69]. Zeta potential values are represented in mV, with zeta potential values within the ±30 mV range usually indicating moderate stability [70]. Gram-positive and Gram-negative bacteria display different components on their cell surface, such as teichoic acid on Gram-positive bacteria [7] and lipopolysaccharides (LPS) on Gram-negative bacteria [9]. The presence of these surface components will affect the electrostatic properties of the associated nanoparticles and their stability. LPS covers most of the cell surface in Gram-negative bacteria which causes the cell surface to be negatively charged, thus it provides an opportunity for nanoparticles to target these cells by exploiting electrostatic interaction. This negative surface charge possessed by Gram-negative bacteria could explain why nanoparticles aggregate on the surface rather than penetrating the prokaryote cell [71]. Snyder and McIntosh proposed that LPS provides a selective barrier by preventing hydrophilic molecules to penetrate the outer surface membrane [72]. In antibiotic-susceptible bacteria lipid soluble antibiotics such as aminoglycosides can easily permeate through the outer membrane. However, in MDR phenotypes the permeability of the bacterial cell wall is altered, such that it prevents hydrophobic antibiotics to enter [73]. Moreover, in Gram-negative bacteria additional pathways exist that can uptake hydrophilic molecules via general diffusion through porins by which water-soluble antibiotics enter [73]. Hence, there is an opportunity for nanoparticles to use this pathway. Since the Gram-negative bacterial cell surface is mostly negatively charged [74] this will dictate how nanoparticles will interact. Many metal nanoparticles are cationic thus, due to such opposite charges the zeta potential of nanoparticles will be vital to the degree to which nanoparticles will be bound to the bacterial cell wall [75]. Halder and colleagues measured the zeta potentials of both *S. aureus* and *E. coli* and found the zeta potential values for their surface charge to be −35.6 mV and −44.2mV, respectively [69]. 

### 5.5. Nanoparticles as Drug Carriers 

**Figure 6:**
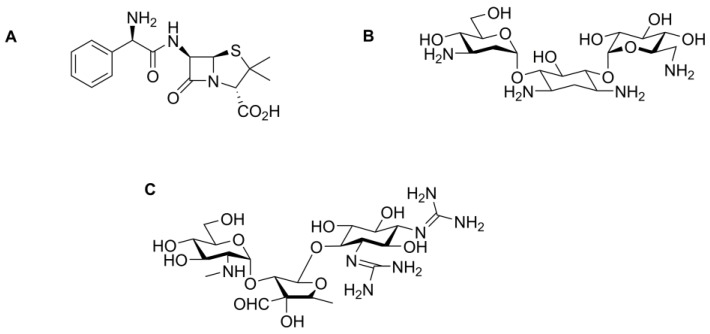
Chemical structures of (**A**) ampicillin; (**B**) kanamycin and (**C**) streptomycin.

The clinical use of conventional antibiotics sometimes requires multiple doses to maintain therapeutic plasma concentrations [76]. Hence, the problem of developing unwanted side effects is increased, especially in elderly patients with impaired renal function which leads to elevated toxicity profiles in the case of potent antibiotics [77]. Furthermore, the problematic physicochemical properties, bioavailability and stability of certain antibiotics can result in the poor therapeutic performance of such antibiotics and may eliminate antibiotic activity altogether. Thus, since the bacteria will be exposed to continual environmental pressure of low concentrations of antibiotics this may potentiate the risk of rising anti-microbial resistance. Conjugating drug molecules onto the surface of nanoparticles can help improve these shortcomings. Hence, numerous studies have emerged reflecting this phenomenon, predominantly using metallic nanoparticles as drug carriers resulting in increased anti-microbial effect. Saha and colleagues conjugated the three different antibiotics ampicillin, kanamycin and streptomycin (Figure 6) onto the surface of colloidal gold [78]. Gold nanoparticles with ampicillin conjugated onto their surface were found to have lower bactericidal activity than gold nanoparticles conjugated with either kanamycin or streptomycin. The authors concluded that the exact reason for this difference in activity profile is not known. However, they hypothesized that the differing bactericidal activity was perhaps due principally to the mechanism of action of the specific antibiotics with the gold nanoparticles attenuating this activity: ampicillin inhibits cell wall formation, whilst kanamycin and streptomycin both inhibit the synthesis of proteins in prokaryotes [78]. 

Brown and colleagues reported the functionalisation of both silver and gold nanoparticles with ampicillin (Figure 6) [79]. They tested the nanoparticles alone as well as the formulated versions against Gram-positive and Gram-negative bacteria. The data showed that the gold nanoparticles only exhibited anti-microbial properties when the ampicillin was bound to the nanoparticle surface, whereas the silver nanoparticles possessed some intrinsic anti-microbial properties, in agreement with other studies presented in the literature. Following conjugation, the novel formulations exhibited a potent effect on a broad range of bacterial species. Importantly, Brown’s study showed that both nanoparticulate formulations exhibited unique properties, which avoided the mechanisms deployed by bacteria that commonly result in the rise of multidrug resistance [79]. Hassan and colleagues reported the use of vancomycin-coated magnetic nanoparticles as anti-bacterial agents [80]. The novel formulation was tested against vancomycin resistant strains, exhibiting minimum inhibitory concentrations as low as 13–28 µg·mL^−1^ compared with 250–4000 µg·mL^−1^ for the free drug. Their nanoparticle formulation rapidly permeated the cell membranes and presented a strategy for re-potentiating drugs [80]. 

Besides metallic nanoparticles, the use of polymers to deliver antibiotics has also been reported. Turos and colleagues reported the development of a polymer-drug conjugate of a poly(acrylate) and an *N*-thiolated beta-lactam drug [81]. The study documented that the nanoparticles formed were 40 nm in diameter and that the preparations showed potent anti-bacterial properties when incubated with methicillin-resistant *S. aureus* compared to the free drug. The authors noted the ease of tailoring of these particles through modification of the acrylate linker that provided conjugation onto the drug molecule [81]. Nguyen and colleagues have developed a sophisticated system in which they modified gentamicin to contain a nitric oxide-releasing moiety [82] and the drug-moiety was then encapsulated within a polymeric nanoparticle. The nanoparticle showed a synergistic antibacterial effect against *P. aeruginosa* of up to 95% when the nitric oxide and drug were released simultaneously. In comparison, administration of the drug alone or nitric oxide alone resulted in only a 20% reduction of bacterial viability [82].

### 5.6. Nanoparticle Biocompatibility 

**Figure 7:**
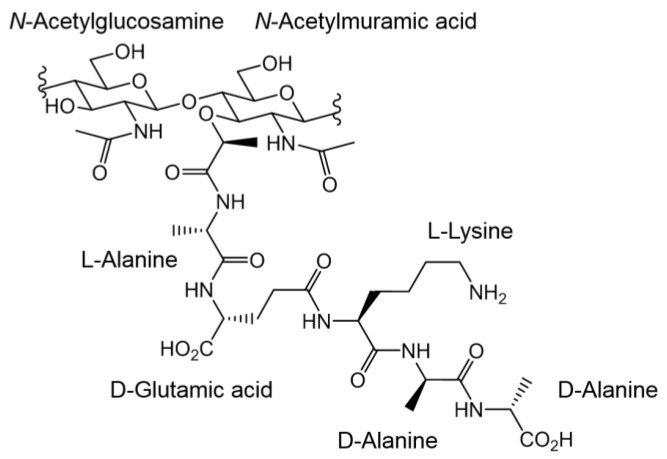
Chemical structure of the peptidoglycan precursor in Gram-positive cells.

As most nanoparticulates are semi- or totally synthetic it is vital that the in vivo toxicity is as low as possible, especially if the nanoparticle transports an antibiotic in a formulation that may affect the normal mechanism and metabolism of the antibiotic. Furthermore, with the utilisation of polymers combined with antibiotics, this may decrease the toxicity profile of the antibiotic which will allow safer usage of these drugs. The mode of action of vancomycin (Figure 4) is via binding to terminal D-Ala-D-Ala residues in precursors of the Gram-positive bacterial peptidoglycan cell wall component [83], preventing formation of the peptide crosslinks which rigidify peptidoglycan (Figure 7). In the case of vancomycin-resistant bacteria the D-Ala-D-Ala pendant is mutated into D-Ala-D-Lactate, as is the case in VanA type resistance, or into D-Ala-D-Ser in VanB type resistance [84]. The genes required for VanA type resistance are found on the transposable DNA fragment Tn1546, usually found on a plasmid or within the bacterial genome [85]. The plasmid DNA is transferred between bacterial cells by conjugation of the cells. The cells which acquire genetic information in this way will now contain the required genes which code for VanA type resistance. Resistance to vancomycin is predominantly observed in *Enterococcus faecium* where the presence of the requisite transposon Tn1546 (containing the *vanA* gene) or Tn1547 (containing the *vanB* gene) will give rise to the incorporation of D-Ala-D-Lactate or D-Ala-D-Ser into the cell wall precursors, respectively, respectively [85]. Acquired resistance to vancomycin in *S. aureus* (VRSA) due to incorporation of transposon Tn1546 into the *S. aureus* genome is thankfully rare but may present a significant challenge [86]. Chakraborty and colleagues showed that vancomycin conjugated to chitosan nanoparticles which were tagged with folic acid showed anti-microbial activity against VRSA [87]. They explained the anti-bacterial action of the folic acid by the polymer acting as the trojan horse which also allowed vancomycin to enter bacteria more readily [87]. Moreover, using chitosan nanoparticles in the conjugated polymer provided biocompatibility that reduced the toxicity profile of the polymer conjugate. Similarly, poly(lactic-*co*-glycolic acid) (PLGA) nanoparticles, of diameters within a range 241–358 nm, entrapping gentamycin were found to act against *P. aeruginosa* [88]. Here, the PLGA nanoparticles allowed controlled release of gentamycin. Similar to chitosan, PLGA is clinically approved and is biocompatible which facilitates usage in vivo [88]. 

**Figure 8:**
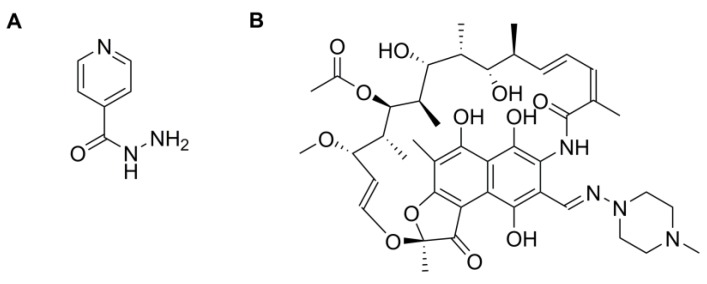
Chemical structures of (**A**) isoniazid and (**B**) rifampicin.

Similarly, PLGA nanoparticles have shown use as a drug carrier for anti-tuberculosis drugs. Tuberculosis (TB) is a serious pathophysiological condition derived from the presence of *M. tuberculosis* bacteria, usually in the respiratory tract [89]. There are several antibiotics available to treat TB that are reserved as first line therapies; however, resistance is also associated with their usage. Examples of the drugs used in first line therapy are isoniazid and rifampicin (Figure 8). In terms of their physiochemical properties, both drugs are lipophilic which affects their absorption when taken orally. Furthermore, there is difficulty in maintaining therapeutic plasma concentrations of the drugs which usually requires several doses to be taken daily. Consequently, side effects occur more frequently. Kalluru and colleagues highlighted the use of PLGA nanoparticles loaded with rifampicin against *M. tuberculosis* [90]. As mentioned earlier, it is difficult to maintain a therapeutic plasma concentration but Kalluru showed that with PLGA nanoparticles of diameter 228 nm loaded with rifampicin therapeutic plasma concentrations were observed up to 12 days after injection in mice [90]. This indicates that the application of PLGA can greatly improve plasma concentrations of rifampicin and its counterparts. This promising in vivo study could potentially be translated into human studies. 

## 6. Conclusions and Future Perspectives

**Figure 9:**
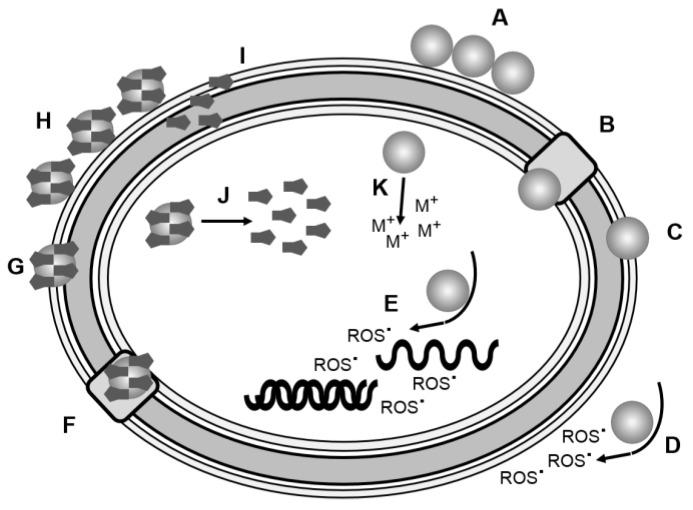
Mechanisms by which nanoparticles can exert an antimicrobial effect: (**A**)Accumulation at the cell wall surface; (**B**) Penetration of the cell, either through damaged cell wall or (**C**) endocytosis; (**D**) Generation of reactive oxygen species externally or (**E**) internally; (**F**) Penetration of nanoparticle and drug payload through damaged cell wall or (**G**) endocytosis; (**H**) Accumulation of nanoparticle and drug payload on cell wall; (**I**) Release of drug payload externally or (**J**) internally; (**K**) Generation of toxic metal ionic species.

Anti-microbial resistance has changed the way antibiotics are used in today’s medicine, requiring clinicians to focus increasingly on changing how antibiotics can be obtained and prescribed. There has been little innovation to discover novel antibiotics that will achieve anti-bacterial action via mechanisms which will be less prone to resistance. Nanotechnology is being exploited to solve complex issues in biomedicine. There is tangible evidence for the effective use of nanotechnologies as anti-microbial agents. Various strategies for the use of nanoparticles in antibiotic chemotherapy have been evaluated, summarised in Figure 9.

Thus, nanoparticle technologies have joined the armamentarium available in the combat against anti-microbial resistance. Resistance to current antibiotics in bacteria could be ameliorated using nanoparticle therapies as evidenced by existing studies. The growing evidence of nanoparticle technologies showing anti-bacterial activity against an array of different pathogens shows a promising future for the application of nanotechnology despite most of the evidence being obtained from in vitro experiments. For nanotechnology to be at the forefront in the future development of modern anti-microbial drugs further experiments will have to undertaken in vivo, especially involving human subjects in clinical trial. Efficacy needs to be proven in humans since therapeutic doses may well be accompanied by intolerable side effects that could inhibit the application of nanoparticles. This is especially important for nanoparticles whose mode of action relies upon the production of ROS: The prokaryotic cells of bacteria are not as complex as eukaryotic cells, which contain structures and organelles that have vital roles in their biology. Ideally, the anti-bacterial action of species which generate ROS would be specific to bacteria. However, eukaryotic cells can also be affected by ROS which will have a detrimental impact on the host. Therefore, therapeutic considerations are vital and empirical evidence is essential for nanoparticle therapies to be applicable in humans. Nevertheless, the use of currently available antibiotics conjugated or associated with nanoparticles brings an alternative dimension to antibiotic therapy: dual anti-bacterial modality using nanoparticles can facilitate treatment and limit the development and impact of anti-microbial resistance. The size and charged nature of nanoparticles can be exploited in the formation of antibiotic drug conjugates. Furthermore, the physiochemical properties of nanoparticles give rise to additional options to deliver a lethal effect to bacteria. In conclusion, there is tangible evidence for the application of nanotechnology in anti-microbial therapies to combat the growing threat of resistance to antibiotics displayed by pathogenic bacteria.

## Figures and Tables

**Table 1:** Examples of nanoparticles exhibiting anti-bacterial properties and their mechanism of action.

Bacterial Species	Gram Stain of the Species	Nanoparticle(s) with Anti-Microbial Activity	Mechanism of Action	Reference
*Staphylococcus aureus*	Gram-positive	Ag	Cell wall damage	[46]
*Campylobacter jejuni*	Gram-negative	ZnO	Growth inhibition by ROS	[47]
*Escherichia coli*	Gram-negative	Cu IONPs	Growth inhibition by ROS ROS generation	[48,49]
*Pseudomonas aeruginosa*	Gram-negative	Ag Zn	Enzyme inhibition in respiratory chain complex ROS generation	[43,50]
*Klebsiella pneumoniae*	Gram-negative	ZnO	Growth inhibition	[42]
*Bacillus subtilis*	Gram-positive	Ag IONPs	DNA degradation ROS generation	[49,51]
